# Development of Paper-Based Fluorescent Molecularly Imprinted Polymer Sensor for Rapid Detection of Lumpy Skin Disease Virus

**DOI:** 10.3390/molecules29071676

**Published:** 2024-04-08

**Authors:** Samr Kassem, Mervat E. Hamdy, Karim M. Selim, Dalia M. A. Elmasry, Momtaz A. Shahein, Dalia M. El-Husseini

**Affiliations:** 1 Nanomaterials Research and Synthesis Unit, Animal Health Research Institute (AHRI), Agricultural Research Center (ARC), Giza 12618, Egypt; 2 Genome Research Unit, Animal Health Research Institute (AHRI), Agricultural Research Center (ARC), Giza 12618, Egypt; 3 Reference Laboratory for Veterinary Quality Control on Poultry Production, Animal Health Research Institute (AHRI), Agricultural Research Center (ARC), Giza 12618, Egypt; 4 Virology Research Department, Animal Health Research Institute (AHRI), Agricultural Research Center (ARC), Giza 12618, Egypt

**Keywords:** LSDV, paper-based, fluorescent, MIPs, biosensor

## Abstract

Lumpy Skin Disease (LSD) is a notifiable viral disease caused by Lumpy Skin Disease virus (LSDV). It is usually associated with high economic losses, including a loss of productivity, infertility, and death. LSDV shares genetic and antigenic similarities with Sheep pox virus (SPV) and Goat pox (GPV) virus. Hence, the LSDV traditional diagnostic tools faced many limitations regarding sensitivity, specificity, and cross-reactivity. Herein, we fabricated a paper-based turn-on fluorescent Molecularly Imprinted Polymer (MIP) sensor for the rapid detection of LSDV. The LSDV-MIPs sensor showed strong fluorescent intensity signal enhancement in response to the presence of the virus within minutes. Our sensor showed a limit of detection of 10^1^ log_10_ TCID50/mL. Moreover, it showed significantly higher specificity to LSDV relative to other viruses, especially SPV. To our knowledge, this is the first record of a paper-based rapid detection test for LSDV depending on fluorescent turn-on behavior.

## 1. Introduction

The virus LSDV, which causes LSD, is a poxvirus homolog that encodes for 30 structural and nonstructural proteins. LSDV infects a wide range of animals including cattle, buffalo, and wild ruminants [1,2,3]. The World Organization for Animal Health (OIE) has classified LSDV as a notifiable contagious illness because of its potential for rapid dissemination and economic effects [4]. It causes temporary or permanent loss in milk production and condition, infertility, abortion, and permanent damage to hides [5,6], resulting in severe financial losses in the affected countries. Restrictions on animal movement, the expense of vaccinations, and the price of treating secondary bacterial infections all contribute to the economic loss [7]. The disease cause morbidity rate can be as severe as 100% [8,9], with a death rate of less than 10%. The most common hosts for this disease are bovines (Bos taurus and Bos indicus) and water buffalo (Bubalus bubalis). Some zoo animals are also susceptible to the infection [10,11,12,13,14], including giraffes, impalas, wildebeest, springboks, and oryxes. Most of Africa suffered from a widespread LSDV epidemic. It has been spreading swiftly in the Middle East and Southeast Europe since 2013. Additionally, multiple epidemic reports have been recorded in Southeast Asian nations since 2019 [10,15].

Quarantine restrictions are difficult to implement, as it was reported that LSDV can be viable for an additional 120 days in infected tissues [16]. To effectively control or eradicate LSDV in endemic and non-endemic countries, rapid and reliable diagnostic tools are required to achieve a presumptive diagnosis. Virus isolation (VI), fluorescent antibody tests, electron microscopy, polymerase chain reaction (PCR), virus neutralization tests, and enzyme-linked immunosorbent assays are some of the common laboratory techniques used to diagnose LSDV, as outlined in the OIE Terrestrial Manual [17]. Cross-reactivity between *Parapoxvirus* and *Capripoxviridae* in serological assays has resulted in limited specificity [18]. The genotyping and phylogenetic study of LSDV and other capripox viruses have been performed using molecular PCR techniques directed at the P32, RPO30, GPCRs, and ORF103 genes [19,20,21,22].

Recently, biosensors emerged as an attractive solution for quick and accurate infectious diseases diagnosis due to their simplicity, potential for downsizing, and capability for real-time analysis [23,24,25,26,27,28]. The definition of a biosensor that is most frequently used is “an analytical device that includes a biologically active element (or components) in close contact with an appropriate physicochemical transducer to generate a measurable signal directly proportional to the concentration of target substance(s) in the sample” [29,30,31]. A standard biosensor is made up of three components: an element for signal amplification and processing, an element for signal acquisition (electrical, optical, or thermal), and an element for biological recognition (enzyme, antibody, DNA, etc.) with an affinity for the target structure. Remarkably, nanotechnology-based biosensors exhibited great potential with high specificity and sensitivity for the analyzed target [32].

Nano-biosensors usually exploit the chemical, electrical, optical, and magnetic properties of materials, in the best interest of the target to be detected, with a high accuracy and in a time-efficient manner [33,34]. To achieve this goal, nanomaterials and nanostructures, including carbon nanotubes, graphene quantum dots (GQDs), metal oxide nanoparticles (NPs), metal nanoclusters, plasmonic nanomaterials, polymer nanocomposites, and nanogels, were studied and evaluated [35,36] for various viral agents, such as Avian influenza virus [37], orbivirus [38], foot-and-mouth disease viruses [39], and bovine respiratory syncytial viruses [40]. Owing to their biocompatibility, structural compatibility, and high adsorption capacity, nanomaterials have proven to be useful in biosensing applications, improving performance with higher sensitivities and lower detection limits [41]. 

To achieve highly selective recognition sites within a polymeric network, molecular imprinting has proven to be a reliable and attractive method because of its longevity and cost-effectiveness [42]. Although molecularly imprinted polymers (MIPs) have several potential uses, their use in sensing devices has caught researchers’ attention. The selectivity and low cost of biomimetic recognition elements are two of MIPs’ most impressive features [42]. There have been significant advances in the MIP field due to the urgent need to enhance disease diagnostics and therapeutics. While challenges remain, imprinting methods for viral recognition hold great promise as potential novel sensing materials. This thought originates from the highly stable binding phenomena that occur naturally at the molecular level in biological systems [42]. MIPs can be integrated with numerous transducer techniques in a wide range of sensor platforms. MIP-based biosensors could be used for sensitive, rapid, and low-cost point-of-care diagnosis [43].

There are several interesting cases of MIPs synthesized by using whole viruses as templates, and then incorporating those MIPs into various devices. MIP nanoparticles coupled to surface plasmon resonance detection has been reported as a novel MIP technology for the selective and sensitive recognition of Adenovirus [44]. Another group of researchers used MIPs for the diagnosis of each influenza A subtype and evaluated sensor characteristics using a QCM [45]; others developed an intriguing work that used a molecular imprinting strategy as a screening protocol for various influenza A subtypes (H5N1, H5N3, H1N1, H3N2, and H6N1) [46]. Also, the electrochemical polymerization of the oxidized o-aminophenol film was utilized with FMDV serotype O on a gold screen-printed electrode [47].

Paper material is a great carrier for developing quick detection technology in different fields [48] due to its low cost, simple transportation, good capillary force, environmentally benign nature, and good biocompatibility. In addition, the coffee ring phenomenon occurs when a liquid is dropped on paper, depending on the capillary force and liquid evaporation [49], and this property can be exploited for target enrichment to enhance detection sensitivity [50].

Herein, we present a nitrocellulose paper-based fluorescent MIPs sensor for the rapid detection of LSDV with high selectivity, sensitivity, and specificity. To the best of our knowledge, a turn-on fluorescence assay using an MIP paper-based system has not been reported before for any animal virus. 

## 2. Results and Discussion

MIPs are a category of synthetic polymers that have been engineered with the purpose of exhibiting selective recognition and binding capabilities towards particular target molecules. The synthesis of these polymers occurs by a technique referred to as molecular imprinting, wherein template molecules are integrated into the polymer matrix during a process called polymerization. The polymerization protocols utilized in molecular imprinting require the precise choice of monomers, cross-linkers, and template molecules. The procedure commonly starts with the establishment of a complex between the template and functional monomers, followed by polymerization to create the final product of a specific imprinted polymer. After the removal of the template molecules, the polymer retains cavities that possess binding sites with molecular specificity. The inclusion of negative controls is crucial in order to ascertain that the selectivity seen in the final MIP is solely attributable to the molecular imprinting process, rather than being influenced by the polymer matrix itself, hence playing a crucial role in confirming the specificity of molecular recognition towards the template molecule, while ensuring that any observed interactions are not due to nonspecific binding or inherent characteristics of the polymer structure. The inclusion of negative controls enables researchers to assess and confirm the efficacy and dependability of molecularly imprinted polymers across diverse applications. Molecular imprinting methods have countless potential uses and have already been implemented in a variety of contexts, such as molecular sensing, antibody screening, drug administration, and protein/virus classification [46,51]. The templates to be imprinted could be anything from a single molecule to a complex mixture, or a simple protein to a complex structure such as viruses or bacteria [52]. The significance of MIPs lies in their capacity to emulate the molecular recognition mechanisms observed in natural systems. Moreover, they provide several advantageous characteristics, including rapid synthesis, enhanced stability, durability, high selectivity, and cost-effectiveness. Furthermore, they can distinguish and differentiate between very similar molecular systems [53]. 

In our study, this assay depended on imprinting all surface characters, not only size with high selectivity and binding affinity to the template molecule, with specific recognition cavities. MIPs were prepared by the polymerization assay using a co-polymer mixture consisting of acrylamide, N-Fluorescien acrylamide, methacrylic acid, and N-vinyl pyrrolidone as monomers, and N,N-(1,2-dihydroxyethylene) bisacrylamide as a crosslinker with an adjusted ratio, which directly affect the sensitivity and specificity of the developing sensor [46]. Three methods exist for the preparation of fluorescent MIPs: (1) the addition of fluorescent monomers or crosslinkers by one-pot polymerization, which necessitates the synthesis of special fluorescent molecules for different templates; (2) the introduction of fluorescent molecules by post-imprinting modification, using, e.g., click chemistry due to its mild reaction conditions, high yield, and high reaction selectivity; and (3) the creation of recognition sites on fluorescent nanomaterials [54].

### 2.1. Structure Characterization of LSDV and SPV

**Figure 1:**
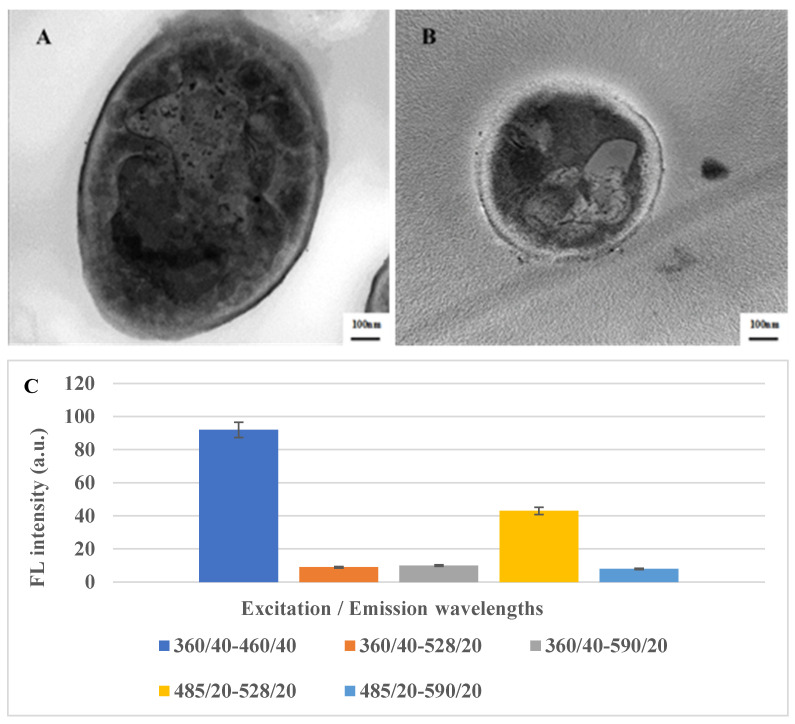
TEM imaging of LSDV (**A**) and SPV (**B**) showing a typical “brick-shaped” morphology with rounded ends and sizes of 290 and 260 nm, respectively. (**C**) Fluorescence spectra of LSDV at different Excitation/Emission wavelengths.

LSDV and SPV were morphologically investigated using TEM (Figure 1A,B) to compare the morphological characters of both viruses. The results showed that the shape and size of the two viruses are highly similar, presenting a typical enveloped brick-like shape with rounded ends and sizes of 290 and 260 nm, respectively, as reported by the authors of reference [55]. The fluorescence absorption of LSDV was detected at different Excitation/Emission wavelengths (360/40-460/40, 360/40-528/20, 360/40-590/20, 485/20-528/20, 485/20-590/20, respectively). The highest fluorescence intensity was recorded at Excitation/Emission wavelength (360/40-460/40) (Figure 1C). 

### 2.2. Characterization of the NIP and MIP-NCM

**Figure 2:**
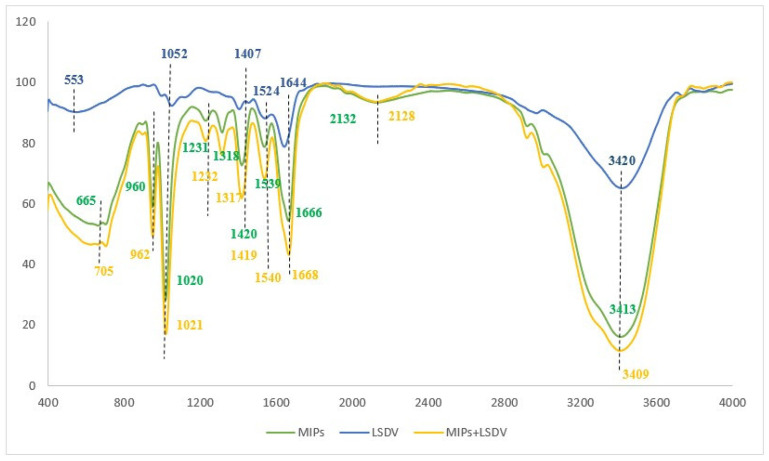
FT-IR spectra of the LSDV, MIPs, and MIPs with LSDV.

After successfully synthesizing the MIP, the chemical composition and involved functional groups in the establishment of the LSDV–MIP complex was evaluated using ATR-FTIR at a scanning range of 4000–400 cm^−^^1^ [45]. Hence, the FTIR spectra were recorded to inspect the chemical functional groups on the surface of LSDV and MIPs before and after binding. The LSDV FTIR results showed characteristic peaks at 3420 cm^−1^ and 1644 cm^−1^, corresponding to the stretching vibrations of the O-H and C=N groups, while the recognized peaks at 1524 cm^−1^ and 1407 cm^−1^ were attributed to the stretching vibrations of the N-O and S=O groups. In addition, the peaks at 1052 cm^−1^ and 553 cm^−1^ were attributed to the stretching vibration of the C-O and C-Cl groups. For MIPs, the FTIR revealed peaks located at 3413 cm^−1^, 2132 cm^−1^, 1666 cm^−1^, and 1539 cm^−1^, which refer to the stretching vibrations of O-H, N=N=N, C=O, and N-O, respectively. Also, peaks were detected at 1420 cm^−1^ and 1318 cm^−1^, indicating the bending vibration of the O-H group. While the peaks detected at 1231 cm^−1^ and 1020 cm^−1^ correspond to the stretching vibration of the C-N groups, likewise, the peak detected at 960 cm^−1^ refers to the bending vibration of the C=C group and the stretching vibration peak of the C-Br group at 665 cm^−1^. The FTIR spectra of the LSDV–MIP complex revealed the shifting of all functional groups of O-H, N=N=N, C=O, and N-O, represented by peaks at 3409 cm^−1^, 2128 cm^−1^, 1668 cm^−1^, and 1540 cm^−1^ with stretching mode, respectively. Also, the shifting of the O-H group, which was detected at 1419 cm^−1^ and 1317 cm^−1^ with bending mode; the peaks at 1232 cm^−1^ and 1021 cm^−1^, which refer to the C-N group with stretching vibration mode; the bending vibration peak of the C=C group, which was detected at 962 cm^−1^; and the bending vibration peak of the C=C group, which was observed at 705 cm^−1^ are shown in Figure 2 and Table 1. The shifted peaks in the FTIR spectra presented in our study prove the success of the formation of the LSDV–MIP complex and the involvement of different functional groups in strengthening the bond between the synthesized MIP and LSDV. 

**Figure 3:**
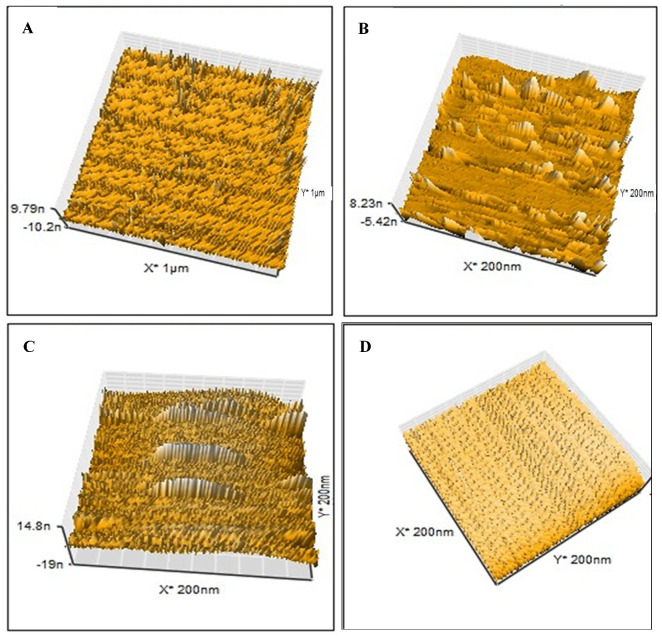
AFM images (3D) of (**A**) the LSD virus used as a template at a scale of 1 µm with sizes ranging from 290 to 295 nm; (**B**) the imprinted bare MIPs following polymerization at a scale of 200 nm; (**C**) LSDV can rebind in the specific sites on the bare MIPs after template removal at a scale of 200 nm. (**D**) NIPs formed without LSDV template virus with smooth surface at scale of 200 nm.

**Figure 4:**
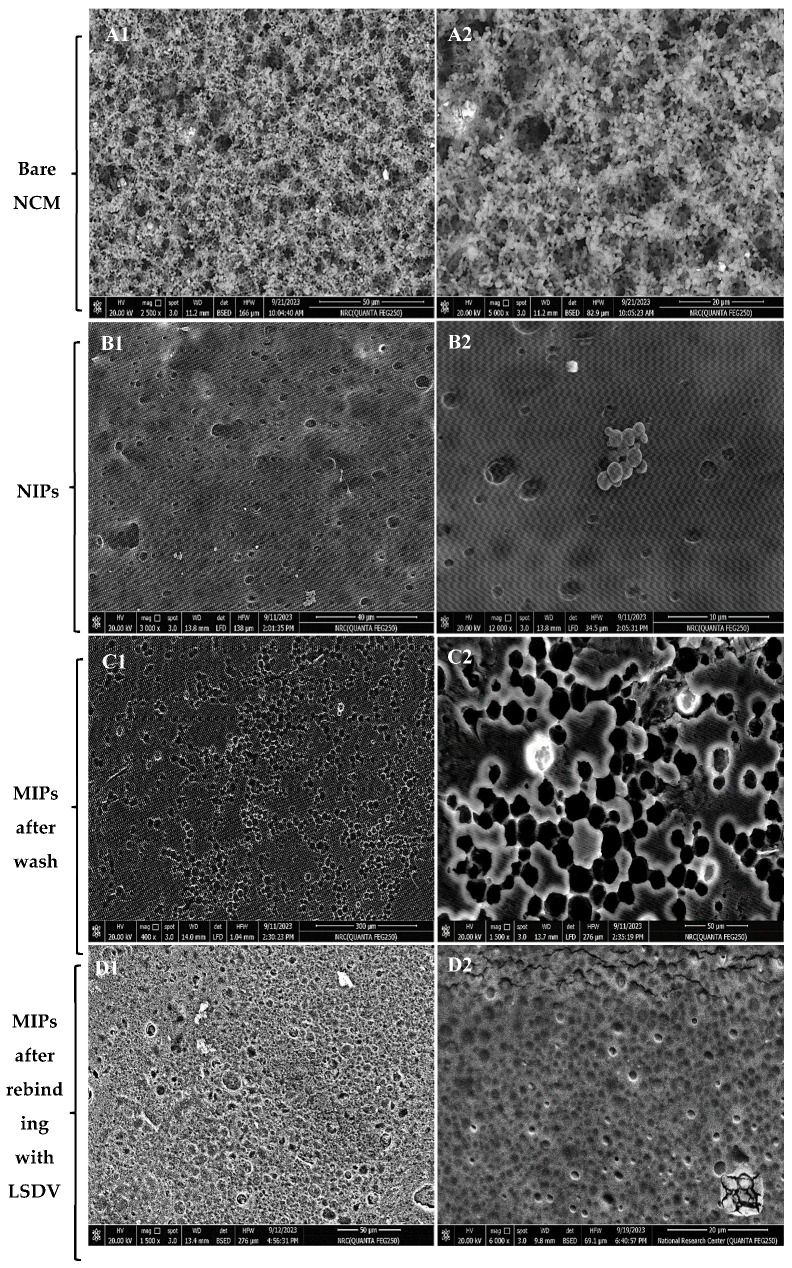
FE-SEM images of (**A1**,**A2**) the bare nitrocellulose membrane, (**B1**,**B2**) NIPs formed without LSDV template virus with homogenous MIPs particles with average size 841.8–868.7 nm, (**C1**,**C2**) MIPs directly after washing with HCL showed holes which were well distributed on the surface of the MIP film with an average size of 449.7 nm–2.34 µm. (**D1**,**D2**) MIPs after rebinding with the LSDV template; the holes appear to be blocked with viral particles, specifically in binding sites.

The LSDV template, NIP, MIP, and MIP–LSDV complex were investigated using AFM to visualize and analyze the topographical features, including the structure, pore size, roughness, and the distribution of imprinted sites on the imprinted polymer layer. AFM (3D) images were recorded for the LSDV template (Figure 3A), MIP (Figure 3B), and MIP–LSDV complex after template removal (Figure 3C). Significantly, the LSDV layer demonstrated a uniform distribution across the surface, exhibiting a roughness of 19.9 nm and diameters ranging between 290 and 295 nm. These dimensions were compatible with those observed via TEM. MIP, following polymerization, resembles the size of the virus template, proving the success of the molecular imprinting process with a height of 8.23 nm. The ability of LSDV to rebind in specific sites on the bare MIP after template removal showed increases in the height of the surface from 8.23 nm and roughness of 0.6 nm, in case of bare MIPs, to 14.8 nm and 1.2 nm, respectively, with grey-colored heights of binding sites in the case of the MIP–LSDV complex, which indicates the success of the binding process. Finally, NIPs formed without LSDV template virus, as shown in Figure 3D, and with a smooth surface. FE-SEM images show the topographical morphology of the bare NCM, and the MIPs after washing with empty cavities, before and after binding with LSDV on the NCM surface, compared with NIPs (Figure 4A–D). MIPs’ thin film arranged homogeneously on the surface of NCM (Figure 4(B1,B2)) with an average size of 841.8–868.7 nm. MIPs, directly after washing with HCL, showed holes which were well distributed on the surface of the MIP film, with an average size of 449.7 nm–2.34 µm (Figure 4(C1,C2)), which may be related to the virus clusters’ agglomerate imprint sites. After rebinding with the LSDV template, the holes appeared to be blocked with viral particles, specifically in binding sites (Figure 4(D1,D2)). AFM and FE-SEM images proved the success of the imprinting process. 

### 2.3. LSDV-MIP Sensor Validation

**Figure 5:**
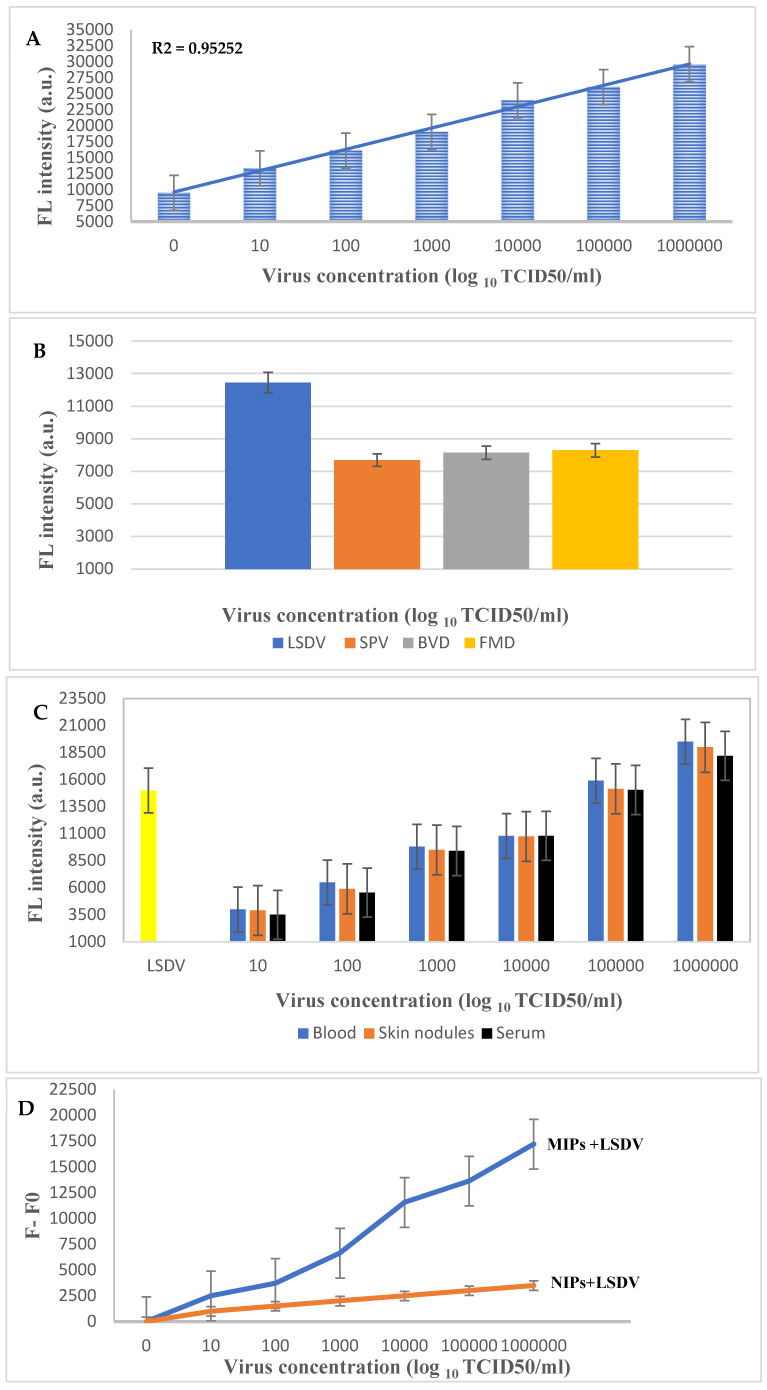
(**A**) Sensitivity test of the developed LSDV-MIPs assay at different concentrations of LSDV (10^1^–10^6^ log_10_ TCID50/mL), (**B**) selectivity test of the LSDV-MIPs assay on SPV, BVDV, and FMDV viruses at a concentration of 10^5^ log_10_ TCID50/mL. (**C**) Application of the LSDV-MIPs assay on real blood, skin nodules, and serum samples spiked with different concentrations of the LSDV template. (**D**) F0 and F represent the fluorescent intensities before and after the addition of the LSDV template, respectively.

To study the sensitivity and recognition ability of the LSDV-MIP sensor, the change in fluorescence intensity in response to the addition of different concentrations of LSDV (10^1^–10^6^ log_10_ TCID50/mL) was measured. Our results revealed a significant enhancement in the fluorescence intensity compared to the negative control (Figure 5A). Moreover, the fluorescence signal increased in direct proportion to the LSDV concentration. The detection limit of the LSDV-MIP sensor was detected to be 1 log_10_ copies/mL under optimal conditions, which is similar to the Real-Time PCR results at CT of 37. Our developed assay was able to detect LSDV with high fluorescence intensity in a short period of time (30 min). This rapid response may occur due to the presence of sufficient empty cavities where LSDV could specifically bind to it. Furthermore, our sensor was able to detect LSDV with the same sensitivity in a complex matrix, whether in spiked or real samples (Figure 5C), representing the fluorescent intensities before (F0) and after (F) the addition of the LSDV template, respectively (Figure 5D). 

Furthermore, the specificity of the LSDV-MIP sensor was evaluated by measuring the change in fluorescence intensity in response to the presence of SPV, FMDV, and BVDV at a concentration of 10*^5^* log_10_ TCID50/mL (Figure 5B). We observed a significant decrease in the fluorescence intensity recorded after the samples’ addition, resulting in a turn-off activity. This action may be due to the inability of these viruses to fit precisely into the empty imprinted cavities because of the absence of the specific functional groups necessary to specifically bind to the selective recognition site on the MIP surface, resulting in weaker or no binding. These results endorse the ability of the sensor developed in this study to be used as a detection and diagnostic tool for LSDV. An MIP sensor can be used as an effective quantitative method for analyzing viral samples [46]. Additionally, it can differentiate between capripoxviridae family members that attain similar morphological characters.

Herein, we employed turn-on fluorescence technology to enhance the sensitivity of our developed sensor. Fluorescence quenching “turn-off” or enhancement “turn-on”, upon template binding, can be employed for template detection in biosensors. However, for turn-off fluorescence sensors, the detection limit and sensitivity may be diminished due to the presence of high background signals. On the other hand, turn-on fluorescence sensors result in a higher signal-to-background ratio, reducing the interference from background fluorescence and leading to sensitivity enhancement. This technique can be improved with a well-designed FRET (Förster resonance energy transfer) system [56,57]. In a turn-on fluorescence assay using MIPs, to prepare the MIPs, the fluorescent molecules must be positioned close to the material surface; otherwise, the distance between the donor and the acceptor would be too long to give an effective FRET [58,59]. Our LSDV-MIP sensor employs the Förster Resonance Energy Transfer (FRET) mechanism. FRET involves the transfer of energy from an excited donor fluorophore to an acceptor fluorophore through dipole–dipole coupling. This occurs when the emission and absorption spectra of the donor and acceptor molecules overlap within a critical distance of, typically, 1–10 nm. In our system, the N-Fluorescien acrylamide, along with the alkyne and azide groups embedded within the MIP structure, act as the acceptor molecules, while the LSDV acts as the donor molecule. Perfect LSDV binding in the specific MIP grooves causes a spatial rearrangement whereby donor and acceptor molecules move into close proximity, initiating FRET. The “turn-on” mechanism usually demonstrates an increase in test sensitivity due to the lower background and higher signal-to-noise ratio compared to the “turn-off”-dependent techniques [45,60]. Further studies should be performed to study the effect of pH conditions or other factors on sensor performance. 

## 3. Materials and Methods

### 3.1. Chemicals, Supplies and Biological Materials

Acrylamide (CAS No.: 79-06-1), methacrylic acid (CAS No.: 79-41-4), methyl methacrylate (CAS No.: 80-62-6), N-vinyl pyrrolidone (CAS No.: 88-12-0), N,N-(1,2-dihydroxyethylene) bisacrylamide (CAS No.: 868-63-3), dimethyl sulfoxide (DMSO) (CAS No.: 67-68-5), 2, 5-BIS (tert-butylperoxy)-2,5-dimethylhexane (CAS No.: 78-63-7), and hydrochloric acid (HCL) (CAS No.: 7647-01-0) were purchased from Sigma-Aldrish (Gillingham, UK). N-Fluorescien acrylamide was prepared according to reference [61]. Deionized water with a resistivity of 18.2 MΩcm was obtained using a Millipore (MilliQ, Burlington, MA, USA) purification system. Chemicals were HPLC-grade and used without more purification. Madin Darby Bovine Kidney (MDBK) cells was obtained from The Egyptian Organization for Biological Products and Vaccine Production (VACSERA), Egypt. Fetal Bovine Serum (FBS), penicillin, streptomycin, and Dulbecco’s Modified Eagle Medium (DMEM) were purchased from Sigma-Aldrish (Saint Louis, MO, USA). LSDV Neethling strain was provided by Vaccine and Serum Research Institute (VSRI), Egypt, while Foot-and-Mouth-Disease Virus (FMDV) serotype O, Bovine Viral Diarrhea Virus (BVDV), and Sheep Pox virus (SPV) were obtained from virology department, Animal Health Research Institute (AHRI), Egypt. Nitrocellulose membrane (HF180MC100- HIFLOW 180 6X30 Membrane) was purchased from Merck Millipore, Darmstadt, Germany. QIAamp DNA Extraction Kit (Qiagen, Catalog# 51306) was purchased from Qiagen (Germantown, MD, USA) and GPS kits for genetic detection of LSDV were purchased from GPS (Madrid, Spain). 

### 3.2. Equipment

Attenuated total Reflectance-Fourier transform infrared spectroscopy (ATR-FTIR) (Perkin Elmer, Seer Green, UK), Atomic force microscope by a Nanosurf C 3000 Atomic force microscope (AFM) (Liestal, Switzerland), and all AFM images were operated in contact mode using a Nanosurf SNL-10 silicon tip, Field emission scanning electron Microscope (FE-SEM) (Quanta FEG250, Brno, Czech Republic), transmission electron Microscope (TEM) (JEOL JEM-1400, Peabody, MA, USA), and BioTek Synergy HTX Multimode Reader (Agilent, Santa Clara, CA, USA).

### 3.3. Virus Propagation

In this experiment, the LSDV Neethling strain served as a template virus. Plaque-purified virus was used to infect MDBK cells at a multiplicity of infection (MOI) of 0.1, and the resulting viral stock was then titrated using MDBK cell cultures. Then, 10% FBS 100 U/mL penicillin and 100 g/mL streptomycin were added to DMEM to cultivate and sustain the MDBK cells. The produced virus was purified and precipitated by high-speed centrifugation at 50,000 rpm/min for 15 min, then lyophilized by freeze drying. The titer of the virus was expressed in log_10_ TCID50 [62]. Morphological characters of LSDV, compared to that of SPV, were detected by TEM.

### 3.4. Fabrication of the Paper-Based Fluorescent MIP Sensor

Molecular imprinting polymers (MIPs) were synthesized according to reference [46] with minor modifications. Briefly, the polymeric mixture was prepared as follows: 9.0 mg of acrylamide and 4.0 mg of N-Fluorescien acrylamide, 10.4 µL of methacrylic acid, 6.4 µL of methyl methacrylate, and 6 µL of N-vinyl pyrrolidone monomer were mixed with 48 mg of N,N-(1,2-dihydroxyethylene) bisacrylamide as a crosslinker. The prepared mixture was dissolved in 300 µL of DMSO containing 2.4 µL of 2, 5-BIS (tert-butylperoxy)-2, 5-dimethylhexane as an initiator. Afterwards, the mixture was subjected to pre-polymerization at 80 °C for 1 h under stirring, then 1 h at room temperature. For LSDV template preparation, the lyophilized LSDV (7 µL of 0.25 mg/µL) was dropped on a sterile glass slide surface and left to dry at room temperature in a clean sterile laminar flow cabinet. A standard sterile paper puncher was used to cut the Nitrocellulose membrane (NCM) into circular pieces with a diameter of 5.5 mm. The prepared NCM pieces were dipped into the prepared pre-polymerization solution, pressed directly onto the dried virus drop, and incubated under UV light with a wave length of 254 nm overnight until a thin polymerized film was observed on the surface of the NCM. For the preparation of the negative control, NCM was dipped in the pre-polymerization solution but pressed onto an empty sterile glass slide. The NCM pressed against the template is called MIPs-NCM, while that pressed against an empty space are called NIPs-NCM. NIPs and MIPs-NCM pieces were washed by immersion in 10% HCL for 10 s to denature and remove the viral molecules, then incubated in deionized water at 45 °C for 2 h under shaking followed by drying on filter paper.

The chemical composition and binding of the functional groups between LSDV and MIPs were evaluated using ATR-FTIR at a scanning range of 4000–400 cm^−1^. The surface morphologies of the NIPs and MIPs NCM were examined using AFM and FE-SEM. 

### 3.5. LSDV-MIP Sensor Validation

The sensitivity of the LSDV-MIP sensor was evaluated based on the fluorescent intensity detected for different LSDV concentrations (10^1^–10^6^ log_10_ TCID50/mL). Briefly, the NIPs and MIPs-NCM were placed into a 96-well black fluorescence microplate followed by the addition of 20 µL of LSDV dilution. The reaction was incubated for 15 min under shaking conditions to allow for the good distribution of the viral particles on the NCM, followed by an extra 15 min incubation without shaking. The fluorescence intensity was measured using a filter-based microplate reader at excitation and emission wavelengths of 485/20 nm and 528/20 nm, respectively. The specificity of this assay was evaluated in the same manner, except for the type of the virus, SPV, FMDV, and BVDV, at a concentration of 10^5^ log_10_ TCID50/mL. For evaluating the efficiency of the sensor to detect LSDV in the complex matrix of real samples, different LSDV concentrations (10^1^–10^6^ log_10_ TCID50/mL) were spiked into the 15 samples of viral free blood buffy coat, serum, and skin nodule lesions. The samples were tested using Real-Time PCR, where the virus DNA was extracted by using the commercial DNA extraction kits. Thermo-cycling of the extracted DNA was performed in a qPCR master mix solution, and the reaction was run for 40 cycles. The measurement was carried out in the same manner as described above. All experiments were carried out in triplicate. 

## 4. Conclusions

MIPs might be thought of as “plastic antibodies” due to their durability and selectivity in rebinding to their target rather than others, including closely related ones; the quality of the interactions between the components of a MIP (template, monomers, cross linker, polymerization initiator, and solvent) strongly affects the efficiency, affinity, and selectivity of the recognition sites. As a result, it is essential to choose the right components for the MIP during its design in order to ensure that it has the necessary characteristics for a certain application. Using fluorescent MIP technology, we have disclosed a new approach to develop a paper-based turn-on fluorescence sensor for the efficient and rapid diagnosis of LSDV. Our sensor exhibited high specificity for LSDV, with a limit of detection of 10^1^ log_10_ TCID50/mL under optimal conditions. The advantages of the turn-on fluorescence test are its high selectivity, rapid response time, and easy manipulation. The findings presented in this study may pave the way for developing a novel, affordable, and economic MIP-based turn-on fluorescence sensor for the detection of other viral and bacterial agents. 

## Figures and Tables

**Table 1:** FT-IR spectra data of the LSDV, NIPs, and MIPs.

Material	Absorption (cm^−1^)	Chemical Bond	Vibration Mode	Functional Group
LSDV	3420 1644 1524 1407 1052 553	O-H C=N N-O S=O C-O C-Cl	stretching stretching stretching stretching stretching stretching	Alcohol Imine Nitro compound Sulfonyl chloride Primary alcohol Halo compound
MIPs	3413 2132 1666 1539 1420 1318 1231 1020 960 665	O-H N=N=N C=O N-O O-H O-H C-N C-N C=C C-Br	stretching stretching stretching stretching bending bending stretching stretching bending stretching	Alcohol Azide Conjugated ketone Nitro compound Alcohol Phenol Amine Amine Alkyne Halo compound
MIPs and LSDV	3409 2128 1668 1540 1419 1317 1232 1021 962 705	O-H N=N=N C=O N-O O-H O-H C-N C-N C=C C=C	stretching stretching stretching stretching bending bending stretching stretching bending bending	Alcohol Azide Conjugated ketone Nitro compound Alcohol Phenol Amine Amine Alkyne Alkyne

## Data Availability

The data presented in this study are available on request from the corresponding author.
